# Prognostic Role of Tumor Immune Microenvironment in Pleural Epithelioid Mesothelioma

**DOI:** 10.3389/fonc.2022.870352

**Published:** 2022-06-20

**Authors:** Hely Ollila, Mikko I. Mäyränpää, Lassi Paavolainen, Juuso Paajanen, Katja Välimäki, Eva Sutinen, Henrik Wolff, Jari Räsänen, Olli Kallioniemi, Marjukka Myllärniemi, Ilkka Ilonen, Teijo Pellinen

**Affiliations:** ^1^ Institute for Molecular Medicine Finland (FIMM), Helsinki Institute of Life Science (HiLIFE), University of Helsinki, Helsinki, Finland; ^2^ Individualized Drug Therapy Research Program, Faculty of Medicine, University of Helsinki, Helsinki, Finland; ^3^ Department of Pulmonary Medicine, Heart and Lung Center, University of Helsinki and Helsinki University Hospital, Helsinki, Finland; ^4^ Department of Pathology, University of Helsinki and Helsinki University Hospital, Helsinki, Finland; ^5^ Laboratory of Pathology, Finnish Institute of Occupational Health, Helsinki, Finland; ^6^ Department of General Thoracic and Esophageal Surgery, Heart and Lung Center, University of Helsinki and Helsinki University Hospital, Helsinki, Finland

**Keywords:** pleural mesothelioma, tumor immune microenvironment, multiplexed fluorescence immunohistochemistry, prognosis, dendritic cells

## Abstract

**Background:**

Pleural mesothelioma (MPM) is an aggressive malignancy with an average patient survival of only 10 months. Interestingly, about 5%–10% of the patients survive remarkably longer. Prior studies have suggested that the tumor immune microenvironment (TIME) has potential prognostic value in MPM. We hypothesized that high-resolution single-cell spatial profiling of the TIME would make it possible to identify subpopulations of patients with long survival and identify immunophenotypes for the development of novel treatment strategies.

**Methods:**

We used multiplexed fluorescence immunohistochemistry (mfIHC) and cell-based image analysis to define spatial TIME immunophenotypes in 69 patients with epithelioid MPM (20 patients surviving ≥ 36 months). Five mfIHC panels (altogether 21 antibodies) were used to classify tumor-associated stromal cells and different immune cell populations. Prognostic associations were evaluated using univariate and multivariable Cox regression, as well as combination risk models with area under receiver operating characteristic curve (AUROC) analyses.

**Results:**

We observed that type M2 pro-tumorigenic macrophages (CD163^+^pSTAT1^−^HLA-DRA1^−^) were independently associated with shorter survival, whereas granzyme B^+^ cells and CD11c^+^ cells were independently associated with longer survival. CD11c^+^ cells were the only immunophenotype increasing the AUROC (from 0.67 to 0.84) when added to clinical factors (age, gender, clinical stage, and grade).

**Conclusion:**

High-resolution, deep profiling of TIME in MPM defined subgroups associated with both poor (M2 macrophages) and favorable (granzyme B/CD11c positivity) patient survival. CD11c positivity stood out as the most potential prognostic cell subtype adding prediction power to the clinical factors. These findings help to understand the critical determinants of TIME for risk and therapeutic stratification purposes in MPM.

## 1 Introduction

Pleural mesothelioma (MPM) is an aggressive malignancy arising from mesothelial cells lining the chest cavity and lungs. MPM prognosis is poor with a median survival of 10 months (1). Despite the dismal general prognosis, a small portion of patients with MPM (5%–10%) survive remarkably longer following diagnosis (2, 3). The underlying biology explaining the differential patient survival is yet to be explored. Prior studies have highlighted the potential prognostic role of the tumor immune microenvironment (TIME) in MPM (4, 5). Although immunotherapies have shown beneficial outcomes in MPM in clinical trials (6, 7), biomarkers are needed to guide patient selection for conventional chemotherapy versus novel immunotherapies (8).

The TIME of MPM consists of several immune cell populations including subtypes of macrophages, T and B lymphocytes, natural killer (NK) cells, myeloid-derived suppressor cells (MDSCs), and dendritic cells (DCs). Several of these immune cell populations present in MPM tumor tissue have been shown to associate with overall survival. For example, higher numbers of CD20^+^ B lymphocytes and CD4^+^ or CD8^+^ T lymphocytes have been associated with improved prognosis (9–15), although the data regarding CD8 association with survival are controversial (12, 16). Higher numbers of intratumoral regulatory T lymphocytes and programmed cell death protein 1 (PD-1–), lymphocyte-activation gene 3 (LAG3–), and T cell immunoglobulin and mucin domain-containing protein 3 (TIM3)–expressing tumor-infiltrating (CD4^+^) T lymphocytes have been associated with poor prognosis (17), although TIM3 expression alone has been reported to be an independent prognostic factor for longer survival (11). In macrophages, higher expression of CD163 (M2-like) has been identified to associate with shorter survival, especially in relation to CD68 macrophage marker (M1-like) or in relation to either CD8 or CD20 (9, 18). Further, higher densities of tumor infiltrating CD68^+^ macrophages have been identified to associate with poor prognosis in patients with MPM with non-epithelioid histology (19). Finally, it has been shown that intratumor-infiltrating MDSCs are associated with poorer progression-free survival and overall survival (17).

Given the many controversial findings regarding immunophenotypes and survival in MPM, we aimed to systematically profile and classify immune cell populations in MPM TIME with 21 immune cell–specific antibodies using formalin-fixed paraffin-embedded tissue microarrays including samples from 69 patients with epithelioid MPM. We utilized multiplexed fluorescence immunohistochemistry (mfIHC) and digital cell–based image analysis for analyzing the immune cell populations present in tumor-associated stroma and correlated the expressions with patient survival.

## 2 Materials and Methods

### 2.1 Patients, Clinical Data, and Tissue Microarrays

The clinical characteristics of the study population and specific details regarding the tissue microarrays have been described (20). We excluded patients with biphasic histology (n = 5). The study population consisted of 69 Finnish patients with epithelioid MPM diagnosed between 2000 and 2012. The median survival of the patients was 19 months, and the median age at the time of diagnosis was 66 years. Overall survival of 20 patients (29%) was longer than 36 months, and 57 (83%) of the patients were male. The tissue samples were taken at the time of diagnosis, so the patients had not received any treatment by then. The ethics committee of Helsinki University Hospital approved the study (HUS/1057/2019).

### 2.2 Multiplexed Fluorescence Immunohistochemistry and Digital Cell–Based Image Analysis

The mfIHC staining, scanning, and digital cell–based image analysis methods have been previously described (20, 21) and were utilized in the current study as described previously. Briefly, we designed and optimized five mfIHC panels (shown in **Supplementary Table S1**) including 21 antibodies targeting immune cell–specific antigens and analyzed the immune cell populations of interest (**Table 1**) from the multichannel images using cell-based image analysis method. We detected all cells in tissue samples using nuclei 4´,6-diamidino-2-phenylindole (DAPI) staining and classified them either as tumoral or stromal cells based on mesothelial staining [cytokeratin 5 (CK5) or a combination of CK5, cytokeratin 5/6 (CK5/6), and calretinin) so that the cells positive for mesothelial markers were defined as tumor cells and remaining cells as stromal cells. Further, we classified the cells either as positive or negative for each specific immune cell marker and marker combinations by using a cutoff for each channel individually based on mean intensity and standard deviation. Finally, we measured the number of immune cells in tumor-associated stroma and calculated the relative amount (%) of cells in the stroma by dividing the number of cells with the total number of stromal cells.

**Table 1:** Immune cell markers and the main marker combinations analyzed in this study.

Main cell population	Marker/marker combination	Specific subpopulation
T lymphocytes	CD3^+^	T lymphocytes
	CD3^+^PD-1^+^	PD-1 expressing T lymphocytes
	CD3^+^PD-L1^+^	PD-L1 expressing T lymphocytes
	CD3^+^PD-1^+^PD-L1^+^	PD-1 and PD-L1 expressing T lymphocytes
	CD3^+^CD8^+^	Cytotoxic T lymphocytes
	CD3^+^CD8^+^PD-L1^+^	PD-L1 expressing cytotoxic T lymphocytes
	CD3^+^CD8^+^PD-1^+^	PD-1 expressing cytotoxic T lymphocytes
	CD8^+^CD11b^+^	CD11b-positive cytotoxic T lymphocytes
	CD8^+^granzyme B^+^	Granzyme B–positive cytotoxic T lymphocytes
	CD8^+^CD11b^+^granzyme B^+^	CD11b and granzyme B–positive cytotoxic T lymphocytes
	CD3^+^IDO^+^	IDO expressing T lymphocytes
	CD3^+^LAG3^+^	LAG3 expressing T lymphocytes
	CD3^+^TIM3^+^	TIM3 expressing T lymphocytes
B lymphocytes	CD20^+^	B lymphocytes
Macrophages	CD11c^+^CD16^+^	
	CD68^+^	M1 macrophages
	CD68^+^pSTAT1^+^	
	CD68^+^HLA-DRA1^+^	
	CD68^+^pSTAT1^+^HLA-DRA1^+^	
	CD163^+^pSTAT1^-^HLA-DRA1^−^	M2 macrophages
	CD163^+^IDO^+^	Exhausted M2 macrophages
	CD163^+^LAG3^+^	
	CD163^+^TIM3^+^	
NK cells	granzyme B^+^	NK cells or cytotoxic T lymphocytes
	granzyme B^+^CD11b^−^	CD11b-negative NK cells or cytotoxic T lymphocytes
	granzyme B^+^CD11b^+^	Granzyme B–positive NK cells
	CD8^+^CD3^−^	
	PD-1^+^CD3^−^	
	PD-1^+^CD3^−^CD8^−^	
DCs	CD11c^+^	Dendritic cells
	CD11c^+^CD16^−^	
Tumor cells	CleavedCaspase3^+^Meso^+^	Cleaved caspase 3–positive tumor cells

NK, natural killer; DCs, dendritic cells; MDSCs, myeloid-derived suppressor cells.

### 2.3 Immune Cell Populations

We investigated the following immune cell populations in MPM tumor tissue: T lymphocytes (including subpopulations expressing checkpoint and exhaustion molecules), B lymphocytes, NK cells, macrophages (including type M1 and type M2 macrophages), DCs, and MDSCs. We identified T lymphocytes using CD3 and CD8 antibodies and detected exhausted T lymphocytes using indoleamine 2,3-dioxygenase (IDO), LAG3, and TIM3 antibodies. For detecting T lymphocytes expressing checkpoint molecules, we used PD-1 and PD-L1 antibodies, respectively. Proliferating T lymphocytes (and other stromal cells and mesothelial tumor cells) were detected using Ki67 antibody. However, because of the high number of Ki67^+^ cells present in many tumor samples, it was difficult to accurately classify as being positive in tumor cells or immune cells. For this reason, we did not include the Ki67 antibody in the analyses. Tumor mitotic count was defined as number of mitoses per 10 high-power field (HPF), and it was calculated using whole tissue sections before constructing the tissue microarrays. CD20 was used for detecting B lymphocytes.

Macrophages and their subpopulations (pro-tumorigenic M2 macrophages and anti-tumorigenic M1 macrophages) were identified using CD68, the transcription factor of musculoaponeurotic fibrosarcoma gene (c-MAF), phosphorylated signal transducer and activator of transcription 1 (pSTAT1), human leukocyte antigen DR alpha 1 (HLA-DRA1) and CD163 antibodies, and NK cells using CD11b, granzyme B, CD56, and CD16 antibodies. In addition, CD11c and CD33 antibodies were utilized for detecting myeloid DCs and cleaved caspase 3 for identifying cleaved caspase 3^+^ tumor cells. The main characterized immune cell populations using specific markers and marker combinations are presented in **Table 1**, and detailed information regarding used antibodies is presented in **Supplementary Table S2**. All characterized immune cell populations are presented in **Supplementary Table S3**. Regarding macrophage markers, it is important to note that CD68 staining was significantly weaker than CD163 staining, and therefore, they were not used in combination.

### 2.4 Statistical Analyses

Statistical analyses were performed using R [R Core Team (2017); R: A language and environment for statistical computing; R Foundation for Statistical Computing, Vienna, Austria] and SPSS (version 25.0, IBM, Armonk, NY, USA). All-cause mortality was used as an outcome measurement. We used univariate Cox regression analysis and continuous values for studying the immune cell populations associated with patient survival. The relative number of specific immune cell types was compared between long- and average-term survivors (cutoff, 36 months) using Mann–Whitney U-test.

Multivariable Cox regression analysis was adjusted for variables associated with survival (p-value of <0.05) in univariate Cox regression and previously known prognostic clinical factors: age, gender, grade, and stage of the disease [defined on the basis of the eight edition of the TNM and further dichotomized into low (IA, IB, and II) and high (IIIA, IIIB, and IV) groups] (3). Nuclear grading (low- and high-grade groups), were defined on the basis of the 2021 WHO classification of epithelioid mesothelioma by two expert pathologist (MIM and HW) (22–24). The proportional hazards assumption was tested by assessing the relationship between Schoenfeld residuals and time. Continuous data were evaluated for skewness by using histograms. The Spearman’s rank correlation coefficient was used to assess correlation between continuous nonparametric variables.

AUROC [area under receiver operating characteristic (ROC) curve comparison using the DeLong’s test for two correlated ROC curves] analysis was used for studying whether the prognostic immune cell subtypes as single variables may add prognostic value to the clinical variables and further increase confidence in a prediction model. For AUROC analyses, all continuous variables (including immune cell variables and age) were dichotomized using median cutoffs, and the follow-up time was cut to 3 years, as longer time did not increase the confidence when significant (not shown). Novel combination risk scores were also transformed to Kaplan–Meier log rank statistics with full follow-up time.

Survival time was calculated as the time from pathological diagnosis (the date the diagnostic tissue sample was taken) to date of death. Three patients were still alive at the end of follow-up (July 2, 2019).

## 3 Results

### 3.1 Univariate Cox Regression Analysis of Immune Cell Populations

**Figure 1:**
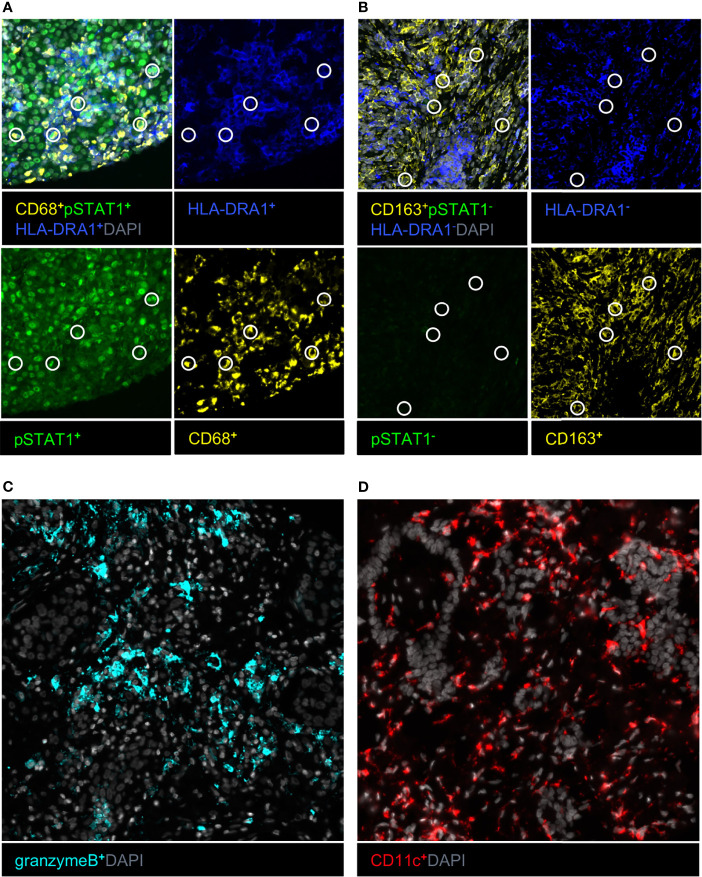
Example images of immune cell phenotypes associated with survival in univariate Cox regression. **(A)** Type M1 macrophage marker combination with circles indicating a specific marker combination. **(B)** Type M2 macrophage marker combination with circles indicating a specific marker combination. **(C)** Granzyme B–positive cells. **(D)** CD11c-positive cells.

The prognostic role of each measured immune cell type (n = 179) present in tumor-associated stroma was studied using univariate Cox regression and continuous values (**Supplementary Table S3**). We found that type M2 macrophages, classified as CD163^+^pSTAT1^−^HLA-DRA1^−^ cells, were associated with shorter survival [hazard ratio (HR) = 1.07; p = 0.002], whereas type M1 macrophages, classified as CD68^+^pSTAT1^+^HLA-DRA1^+^ cells, were associated with longer survival (HR= 0.95; p = 0.033). Granzyme B^+^ cells and CD11c^+^ cells were associated with longer survival (HR = 0.32; p = 0.005, and HR = 0.94; p < 0.001) (**Table 2**). As CD11c is expressed both by DCs and monocytes (25), we analyzed the number of CD11c^+^ cells either positive or negative for a monocyte marker, CD16. In addition, 97.1% of CD11c^+^ cells were negative for CD16 (CD11c^+^CD16^−^), and, respectively, 2.9% of CD11c^+^ cells were positive for CD16 (CD11c^+^CD16^+^), suggesting that the CD11c-associated prognostic effect is largely contributed by DCs. Indeed, CD11c^+^CD16^−^ cells were associated with favorable prognosis, whereas CD11c^+^CD16^+^ did not (**Table 2**). Example images of immune cell phenotypes associated with survival are presented in **Figure 1**. Regarding checkpoint inhibitors (PD-1 and PD-L1) and exhaustion markers (TIM3, LAG3, and IDO), which are targets of immunotherapies and thus are also currently of great interest also in mesothelioma research (26), we did not observe any associations with patient survival (**Supplementary Table S4**) (6, 7).

**Table 2:** Selected immune cell classes and their survival effects (continuous cell classes).

Marker/marker combination	HR	p-value	Prognosis
Granzyme B^+^	0.32	<0.01	Favorable
Granzyme B^+^CD8^+^	1.76	0.55	
Granzyme B^+^CD11b^+^	0.08	0.31	
Granzyme B^+^CD11b^−^	0.32	<0.01	
CD11c^+^	0.94	<0.01	
CD11c^+^CD16^-^	0.93	<0.01	
CD11c^+^CD16^+^	0.88	0.47	
CD68^+^	0.99	0.12	
CD68^+^pSTAT1^+^HLA-DRA1^+^	0.95	0.03	
CD163^+^	1.00	0.97	Unfavorable
CD163^+^pSTAT1^−^HLA-DRA1^−^	1.07	<0.01	

A HR of >1 indicates an increased risk of death, and HR of <1 indicates a decreased risk of death. The relative number of each immune cell type was measured in tumor-associated stroma. All univariate Cox regression results are presented in **Supplementary Table S3**.

HR, hazard ratio.

### 3.2 The Relative Numbers of Prognostic Immune Cell Types Between Average-Term and Long-Term Survivors

**Figure 2:**
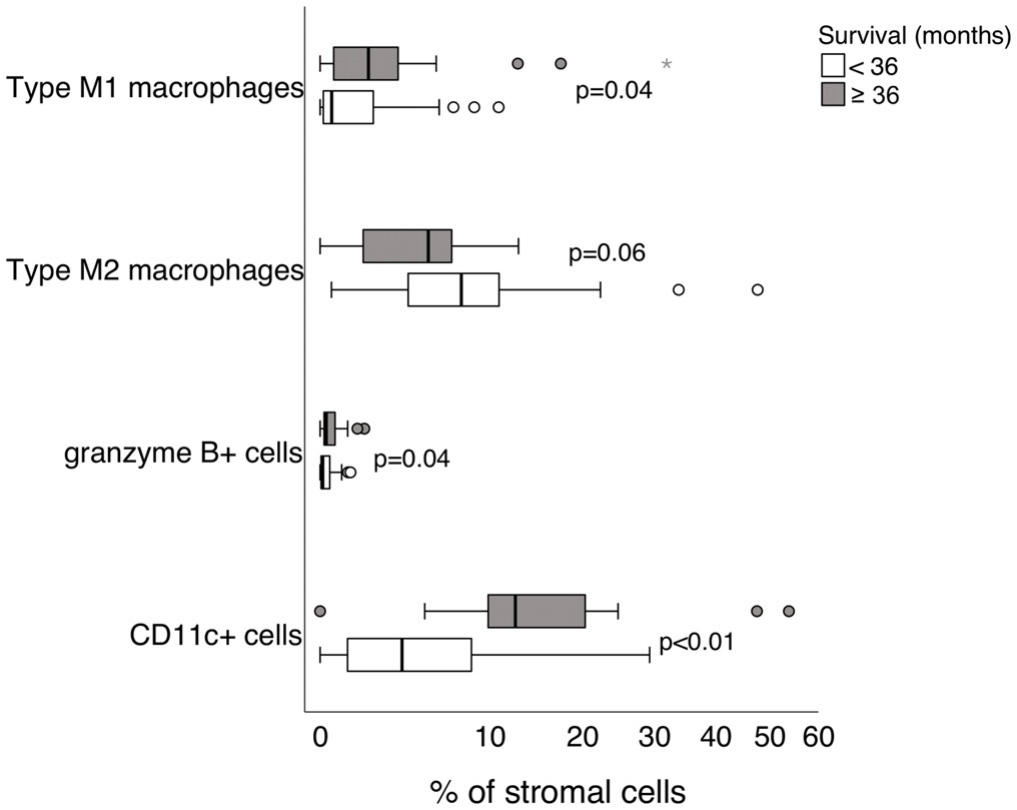
The relative number (%) of each immune cell type of all stromal cells in long-term and average-term survivors (cutoff, 36 months). The cell classes that are significantly associated with survival in univariate Cox regression are shown. Differences between groups were tested by using the Mann–Whitney U-test.

The relative number (percentage of all stromal cells) of each immune cell type was measured, and their distributions in long- and average-term survivors (cutoff, 36 months) were compared. The immune cell phenotypes associating with survival in univariate Cox regression (from **Table 2**) are presented in **Figure 2**. Granzyme B^+^ cells and CD11c^+^ cells were more abundant in long-term survivors, whereas M2-like macrophages were less frequent in long-term survivors (**Figure 2**).

### 3.3 Multivariable Cox Regression and Combination Risk Models

**Figure 3:**
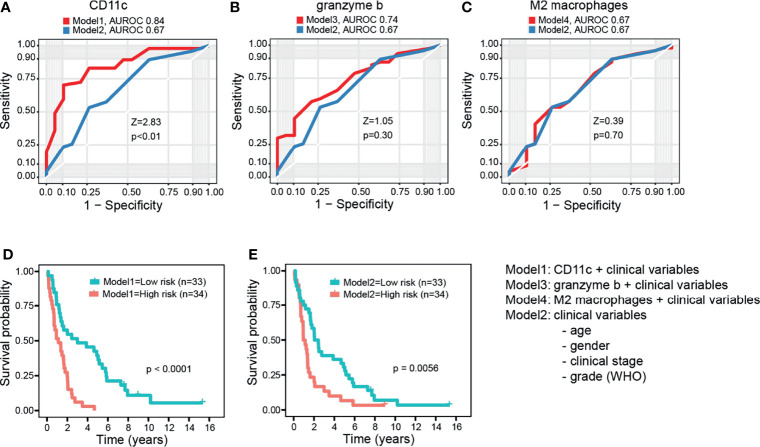
Survival prediction power of combination models. **(A)** Area under the receiver operating characteristic curves (AUROC) for combined clinical factors (age, gender, clinical stage, and grade) (Model2) and for clinical factors plus CD11c (median dichotomized) (Model1). **(B, C)** Same as in **(A)**, but now granzyme B and M2 macrophages in Models 3 and 4, respectively. The DeLong’s test for two correlated ROC curves. **(D, E)** Kaplan–Meier curves for low- and high-risk dichotomized patient groups with and without CD11c in the model, respectively. Log rank test.

In multivariable Cox regression adjusting for age, gender, grade, clinical stage, and each prognostic immune cell type separately, type M2 macrophages, granzyme B^+^ cells, and CD11c^+^ cells remained associated with survival. When combining clinical factors and prognostic immune cell types (type M2 macrophages, granzyme B, and CD11c) in one model, all three immune cell populations remained independently associated with survival. All multivariable Cox regression results are presented in **Table 3**. To assess whether type M2 macrophages, granzyme B, or CD11c as single variables would add power in a prediction model together in combination with the following clinical variables: age, gender, clinical stage, and grade, we performed AUROC comparisons between the models. CD11c was the only immune cell subtype increasing confidence in a prediction model, and therefore, CD11c stood out as the most significant prognostic factor of the immune cell subtypes studied (**Figure 3**).

**Table 3:** Multivariable Cox regression analysis.

Variable	HR (95% CI)	p-value
**Individual markers** (n = 66*)		
CD68^+^pSTAT1^+^HLA-DRA1^+^	0.94 (0.87–1.01)	0.11
CD163^+^pSTAT1^−^HLA-DRA1^−^	1.06 (1.02–1.10)	<0.01
granzyme B^+^	0.32 (0.12–0.87)	0.03
CD11c^+^	0.93 (0.89–0.98)	<0.01
**All markers combined** (n = 66*)		
Age	1.00 (0.97–1.03)	0.88
Gender		
Male	1.0	
Female	0.96 (0.48–1.95)	0.92
Clinical stage		
Low	1.0	
High	1.77 (1.00–3.12)	0.05
Grade		
Low	1.0	
High	1.13 (0.50–2.55)	0.76
CD163^+^pSTAT1^−^HLA-DRA1^−^	1.05 (1.01–1.10)	0.02
granzyme B^+^	0.29 (0.11–0.76)	0.01
CD11c^+^	0.94 (0.90–0.99)	0.01

Multivariable Cox regression adjusted for age, gender, grade, and clinical stage. A HR of >1 indicates an increased risk of death, and a HR of <1 indicates a decreased risk of death.

*TNM staging missing for one patient, univariate Cox regression results missing for 2 patients.

All models fulfilled the proportional hazard assumption.

HR, hazard ratio; CI, confidence interval.

### 3.4 Correlations Between Prognostic Immune Cell Types

Both type M1 and type M2 macrophages correlated positively with Ki67^+^-proliferating cells (Spearman’s rho 0.27, p = 0.03 and 0.34, p = 0.01, respectively), and CD11c^+^ cells correlated negatively with type M2 macrophages (Spearman’s rho −0.32, p = 0.01). Ki67^+^-proliferating cells correlated positively with mitoses (Spearman’s rho 0.30, p = 0.02), whereas there was no correlation between type M2 macrophages and mitoses (Spearman’s rho 0.08, p = 0.54).

## 4 Discussion

In this study, we investigated the prognostic immune cell phenotypes present in tumor-associated stroma of epithelioid mesothelioma. In univariate analysis, we found that type M2 macrophages were associated with shorter survival. Further, type M1 macrophages and CD11c or granzyme B expressing cells were associated with longer survival. When comparing the relative numbers of these cell types between average and long-term survivors (cutoff, 36 months), especially higher numbers of CD11c expressing cells were associated with long-term survival. In multivariable Cox regression adjusting separately for prognostic immune cell phenotypes (from univariate analysis), age, gender, grade, and stage of the disease, all phenotypes excluding type M1 macrophages remained independently associated with survival. When taking into account all prognostic phenotypes, type M2 macrophages, granzyme B, and CD11c remained independently associated with survival. Overall, the most important findings of this study were that M2-like macrophages are independently associated with shorter survival, and granzyme B and CD11c with longer survival in MPM. Finally, CD11c was the only immune cell subtype increasing confidence in a prediction model.

We observed that type M2 macrophages were associated negatively with survival, also when it was adjusted for other immunophenotypes together with clinical factors in a multivariable model. Prior studies have shown that type M2 macrophages increase the proliferation of MPM cells and their resistance to chemotherapy (27). We also observed a positive correlation between type M2 macrophages and Ki67 but not between type M2 macrophages and mitosis. Further, higher CD163/CD8 and CD163/CD20 ratios have been shown to be independent prognostic factors of shorter survival in MPM (9), and patients with MPM with higher M2/total tumor-associated macrophage (TAM) ratio have been shown to be more prone to develop local tumor outgrowth compared with those with low M2/TAM ratio (28). However, a previous study indicated no prognostic value for M2 macrophages, possibly due to median-based cutoff values for the percentage of macrophages, whereas we assessed the survival effect by using continuous values (17). Our findings also demonstrated that CD68^+^ M1 macrophages associate with longer survival, although they were not independent of other factors in a multivariable model. These findings where M2 and M1 macrophages associate with poor and favorable survival, respectively, are logical, as many studies in other cancers, in addition to the aforementioned studies in MPM (9, 27), indicate the same (29). We classified M2 macrophages as CD163^+^pSTAT1^−^HLA-DRA1^−^ cells and M1 macrophages as CD68^+^pSTAT1^+^HLA-DRA1^+^ cells.

Our finding regarding type M2 macrophages supports the previous observations that higher number of them in MPM TIME predicts shorter survival in patients with MPM. Therefore, the number of M2 macrophages in MPM TIME may be used as prognostic tool when prognosticating the survival of patients with MPM. Further, it is known that type M2 macrophages are typically present in Th2-type processes (30, 31), and in tumor immunology, this is usually considered immunologically cold. Such tumors typically do not respond well to immuno-oncological (IO) treatments (32). On the basis of our findings, immunologically hot MPM tumors seem to have better prognosis even without treatment, but they are also likely to respond better when treated. This may be a potential future prospect when targeting IO treatments to patients with MPM.

Granzyme B is known to be expressed by cytotoxic T cells, NK cells, mast cells, plasma cells, and myeloid cells (33–35). Marcq et al. investigated the prognostic value of granzyme B in patients with pretreated and unpretreated MPM but reported no association between granzyme B expression and patient survival (11). Further, Mankor et al. reported that patients with MPM who were responding to aPD-1/aCTLA-4 combination treatment (nivolumab combined with ipilimumab) had a higher amount of granzyme B expressing CD8^+^ cytotoxic T cells (36). We investigated the prognostic value of granzyme B alone as well as in combination with CD8 (marker for cytotoxic T cells) or with CD11b (marker for myeloid cells). Marker combinations were not of prognostic value, but we showed that granzyme B alone is an independent positive prognostic factor in MPM. Therefore, granzyme B could be used for prognostication of survival, and it remains to be explored if it could potentially also be a marker for targeting IO treatments (as aPD-1/aCTLA-4 combination treatment) to a more specific subgroup of patients.

CD11c is expressed by the cells of myeloid origin and is typically used as a marker of differentiated DCs but may also be expressed by CD16^+^ monocytes and macrophages. DCs are key antigen-presenting cells, important in mediating immune activation in the tumor microenvironment of several types of malignancies (25). Expression of DCs is also proposed to result in a loss of function of CD4 and CD8 T cells (37). Here, we observed that higher numbers of CD11c^+^ cells predicted longer survival, also when adjusting for other prognostic factors including other immune cell types. We postulate that the prognostic CD11c^+^ cells are DCs, as the double-positive phenotype with the monocyte/macrophage marker, CD16, represented only a small fraction of CD11c^+^ cells (2.9%) and was not of prognostic value. CD11c^+^ DCs stood out as the most significant prognostic immune cell subtype, as demonstrated by combination survival prediction models using AUROC analyses. Therefore, we suggest that CD11c alone or with clinical factors could be used as a prognostic marker for predicting the survival of patients with MPM.

### 4.1 Strengths and Limitations

Although the prognostic values of different immune cell markers in MPM TIME have been investigated before, we utilized methodology enabling deep simultaneous quantitative examination of many immune cell types in a unique cohort of patients with MPM including rare long-term survivors. This allowed us to characterize the specific prognostic immune cell populations in a systematic manner and compare the TIME landscape between average and long-term survivors. Because MPM is a rare malignancy, it will be necessary to confirm the data in larger independent cohorts of patients. Our study population included only patients with MPM with epithelioid histology, so the results may not apply to other histological subtypes of MPM.

## 5 Conclusions

After univariate and multivariable analyses, we found that higher number of M2 macrophages in MPM stroma is an independent prognostic factor for shorter survival. In contrast, higher numbers of CD11c^+^ stromal cells and granzyme B^+^ stromal cells in MPM are independent prognostic factors for longer survival. These specific cell types (especially CD11c expressing DCs) may potentially be used as prognostic factors when estimating the risk of disease progression in patients with MPM. Further, these findings warrant for further studies to ask if they could be used to guide targeting of IO treatments to specific subgroups of patients with MPM.

## Data Availability Statement

The raw data supporting the conclusions of this article will be made available by the authors, without undue reservation.

## Ethics Statement

The studies involving human participants were reviewed and approved by the Ethics Committee of Helsinki University Hospital (HUS/1057/2019). Written informed consent for participation was not required for this study in accordance with the national legislation and the institutional requirements.

## Author Contributions

HO analyzed the data, performed the statistical analyses, and wrote the paper. LP developed the scripts for conducting the cell-based image analysis. JP collected the clinical data. HW and ES gathered the study cohort and were responsible for constructing the tissue microarrays (TMAs). KV coordinated and carried out the mfIHC experiments. TP designed the mfIHC panels. OK, MM, and JR provided their special expertise for designing and conducting the study. II, MIM, and TP were responsible for the study design and supervised the project. All authors contributed to the article and approved the submitted version.

## Funding

This study was supported by a special governmental subsidy for health and science research from the Helsinki University Hospital, The Finnish Work Environment Fund, Svenska kulturfonden, K. Albin Johansson stiftelse, Finska Läkaresällskapet, Mjölbolsta stiftelse för medicinsk forskning, Medicinska Understödsföreningen Liv och Hälsa, The Finnish Medical Foundation, Sigrid Jusélius Foundation, Tampereen tuberkuloosisäätiö, Väinö ja Laina Kiven Säätiö, and Hengityssairauksien tutkimussäätiö.

## Conflict of Interest

The authors declare that the research was conducted in the absence of any commercial or financial relationships that could be construed as a potential conflict of interest.

## Publisher’s Note

All claims expressed in this article are solely those of the authors and do not necessarily represent those of their affiliated organizations, or those of the publisher, the editors and the reviewers. Any product that may be evaluated in this article, or claim that may be made by its manufacturer, is not guaranteed or endorsed by the publisher.

## Abbreviations

MPM, pleural mesothelioma; TIME, tumor immune microenvironment; NK, natural killer; MDSCs, myeloid-derived suppressor cells; DCs, dendritic cells; mfIHC, multiplexed fluorescence immunohistochemistry; CK5, cytokeratin 5; CK5/6, cytokeratin 5/6; c-MAF, transcription factor of musculoaponeurotic fibrosarcoma gene; pSTAT1, phospho-STAT1; TNM, tumor, nodes, metastases; TAM, tumor-associated macrophage; IO, immuno-oncological; AUROC, area under receiver operating characteristic curve.
