# Supplementary material for: Prognostic Role of Tumor Immune Microenvironment in Pleural Epithelioid Mesothelioma
Source: Front Oncol. 2022 Jun 20;12:870352. doi: 10.3389/fonc.2022.870352 (PMC9251441; doi:10.3389/fonc.2022.870352)
Supplement: Supplementary file 1 [file DataSheet_1.pdf]

Supplementary Material

1 Supplementary Tables

Table S1. mfIHC antibody panels.

Channel	Panel 1	Panel 2	Panel 3	Panel 4	Panel 5
488	PD-1	CD68	Cleaved Caspase 3	CD11c	IDO
555	PD-L1	c-MAF	CD33	CD3	LAG3
647	CD8	pSTAT1	CD8	CD20	TIM3
750	CD3	HLA-DRA1	CD11b	CD56	CD3
647 (2nd round)	meso	CD163	granzyme B	CD16	CD163
750 (2nd round)	Ki67	meso	meso	meso	meso

Abbreviations: mfIHC, multiplexed fluorescence immunohistochemistry; meso, mesothelial staining

Table S2. Antibody details.

Antibody	Manufacturer	Source code	Clone/polyclonal
CD3	Thermo Fisher Scientific	MA5-14482	EP449E
CD8	Agilent Dako	M7103	C8/144B
PD-1	LSBio™	LS-B12784	3C6
PD-L1	Cell Signaling Technology®	#13684	(E1L3N®) XP®
Ki67	Agilent Dako	M7240	MIB-1
CD68	Agilent Dako	M0876	PG-M1
c-MAF	Abcam	ab199424	EPR16484
phospho-STAT1	Cell Signaling Technology®	#8826	(Ser727) (D3B7)
HLA-DRA1	Abcam	ab20181	TAL 1B5
CD163	Abcam	ab188571	EPR14643
Cleaved Caspase 3	Cell Signaling Technology®	#9661	Asp175
CD33	LSBio™	LS-C338084	OTI2C1
CD11b	BioSB	BSB 6441	EP45
granzyme B	Abcam	ab4059	polyclonal
CD11c	Abcam	ab52632	EP1347Y
CD20	Epredia™	MS340S	L26
CD56	Cell Marque™	MRQ-42	-
CD16	Cell Marque™	SP175	-
IDO	Cell Signaling Technology®	#86630	D5J4E™
LAG3	LSBio™	LS-C18692	17B4
TIM3	Cell Signaling Technology®	#45208	(D5D5R™) XP®
CK5	Abcam	ab128190 / ab52635	2C2 / EP1601Y
calretinin	Agilent Dako	M724529-2	DAK-Calret 1
CK5/6	Cell Marque™	356M-15	D5 & 16B4
DAPI	Roche	1023676001	-

Table S3. Analyzed immune-cell populations and their prognostic effects using continuous values and univariate Cox regression.

Marker/marker combination	HR	*p*-value
CD3-stroma	1.0034	0.7941
CD3+stroma	0.9966	0.7941
PD-1-stroma	0.9995	0.9679
PD-1+stroma	1.0005	0.9679
CD8-stroma	1.0079	0.5962
CD8+stroma	0.9922	0.5962
PD-L1-stroma	0.9971	0.7728
PD-L1+stroma	1.0029	0.7728
CD3+PD-1+stroma	0.9987	0.9355
CD3+CD8+stroma	0.9916	0.6280
CD3+PD-L1+stroma	1.0075	0.7068
CD8+PD-1+stroma	0.9540	0.5485
CD8+PD-L1+stroma	0.9989	0.9583
PD-1+PD-L1+stroma	1.0089	0.6074
CD3+CD8-stroma	1.0081	0.7942
CD3+PD-1-stroma	0.9778	0.5643
CD3+PD-L1-stroma	0.9812	0.3955
CD8+CD3-stroma	0.9664	0.6024
CD8+PD-1-stroma	0.9767	0.5892
CD8+PD-L1-stroma	0.9752	0.3595
CD3-CD8+stroma	0.9664	0.6024
CD3-PD-1+stroma	1.0105	0.7658
CD3-PD-L1+stroma	1.0028	0.8709
PD-1+CD3-stroma	1.0105	0.7658
PD-1+CD8-stroma	1.0264	0.3958
PD-1+PD-L1-stroma	0.9806	0.4800
PD-L1+CD3-stroma	1.0028	0.8709
PD-L1+CD8-stroma	1.0087	0.6050
PD-L1+PD-1-stroma	0.9996	0.9842
CD3+CD8+PD-1+stroma	0.9934	0.7349
CD3+CD8+PD-L1+stroma	1.0016	0.9491
CD3+PD-1+PD-L1+stroma	1.0082	0.7042
CD3+CD8+PD-1-stroma	0.9531	0.4782
CD3+CD8+PD-L1-stroma	0.9636	0.2887
CD3+PD-1+PD-L1-stroma	0.9685	0.4026
CD3+CD8-PD-1+stroma	1.0321	0.5218
CD3+CD8-PD-L1+stroma	1.0825	0.2446

CD3+PD-1-PD-L1+stroma	1.0432	0.8122
CD3-CD8+PD-1+stroma	0.9521	0.5870
CD3-CD8+PD-L1+stroma	0.9446	0.5955
CD3-PD-1+PD-L1+stroma	1.0412	0.4828
CD3-CD8-PD-1+stroma	1.0421	0.4332
CD3-CD8-PD-L1+stroma	1.0054	0.7785
CD3-PD-1-PD-L1+stroma	0.9990	0.9612
CD3-CD8+PD-1-stroma	0.9801	0.8474
CD3-CD8+PD-L1-stroma	0.9782	0.8008
CD3-PD-1+PD-L1-stroma	0.9887	0.8440
CD3+CD8-PD-1-stroma	0.9814	0.7787
CD3+CD8-PD-L1-stroma	0.9869	0.7543
CD3+PD-1-PD-L1-stroma	0.9719	0.4991
CD3+CD8+PD-1+PD-L1+stroma	1.0018	0.9435
CD3+CD8+PD-1-PD-L1-stroma	0.9456	0.4469
CD3+CD8+PD-1+PD-L1-stroma	0.9464	0.2943
CD3+CD8+PD-1-PD-L1+stroma	1.0018	0.9435
CD3+CD8-PD-1-PD-L1-stroma	0.9711	0.6840
CD3+CD8-PD-1+PD-L1-stroma	0.9860	0.8738
CD3+CD8-PD-1+PD-L1+stroma	1.1146	0.1924
CD3+CD8-PD-1-PD-L1+stroma	1.1656	0.6305
CD3-CD8+PD-1-PD-L1-stroma	0.9960	0.9712
CD3-CD8+PD-1+PD-L1-stroma	0.9123	0.6297
CD3-CD8+PD-1+PD-L1+stroma	0.9498	0.6724
CD3-CD8+PD-1-PD-L1+stroma	0.7563	0.5222
CD3-CD8-PD-1-PD-L1-stroma	1.0009	0.9079
CD3-CD8-PD-1+PD-L1-stroma	0.9951	0.9484
CD3-CD8-PD-1+PD-L1+stroma	1.1524	0.1151
CD3-CD8-PD-1-PD-L1+stroma	0.9996	0.9859
CD68+stroma	0.9866	0.1202
CD163+stroma	1.0003	0.9728
pSTAT1+stroma	0.9805	0.0497*
c-MAF+stroma	0.9938	0.5398
HLA-DRA1+stroma	0.9894	0.1814
CD68+pSTAT1+stroma	0.9615	0.0187*
CD68+c-MAF+stroma	0.9655	0.2901
CD68+c-MAF-stroma	0.9874	0.1712
CD68+HLA-DRA1+stroma	0.9753	0.0493*
CD68+pSTAT1+HLA-DRA1+stroma	0.9491	0.0333*
CD163+pSTAT1+stroma	0.9690	0.0790
CD163+c-MAF+stroma	0.9638	0.2227

CD163+c-MAF-stroma	1.0034	0.6845
CD163+HLA-DRA1-stroma	1.0566	0.0142*
CD163+pSTAT1-stroma	1.0115	0.2453
CD163+pSTAT1-HLA-DRA1-stroma	1.0724	0.0024**
CD163+pSTAT1-c-MAF+stroma	0.9819	0.6071
CD163+HLA-DRA1-c-MAF+stroma	0.8297	0.0775
CD163+HLA-DRA1-c-MAF+pSTAT1-stroma	0.8825	0.3539
CD11b+stroma	1.0310	0.3336
CD8+stroma	0.9810	0.5771
CleavedCaspase3+stroma	0.9613	0.2904
CleavedCaspase3+meso	1.1365	0.0652
granzyme B+stroma	0.3181	0.0047**
CD11b+granzyme B+stroma	0.0827	0.3069
CD8+granzyme B+stroma	1.7600	0.5500
CD11b+CD8+stroma	0.8627	0.5638
CD11b+CleavedCaspase3+stroma	1.0862	0.6703
CD11b+granzyme B-stroma	1.0320	0.3217
CD11b+CD8-stroma	1.0382	0.2615
CD11b+CleavedCaspase3-stroma	1.0375	0.3078
CD11b-granzyme B+stroma	0.3200	0.0065**
CD11b-CD8+stroma	0.9817	0.6127
CD11b-CleavedCaspase3+stroma	0.9576	0.2527
CD11b+CD8+granzyme B+stroma	0.0000	0.2147
CD11b+CD8-granzyme B+stroma	0.0823	0.3269
CD11b-CD8+granzyme B+stroma	0.0000	0.0785
CD11c+stroma	0.9407	0.0002***
CD11c+CD16-	0.9335	0.0001***
CD11c+CD16+	0.8843	0.4669
CD3+stroma	1.0082	0.0932
CD20+stroma	1.0023	0.8922
CD16+stroma	1.0080	0.9232
CD16+CD3-stroma	0.9926	0.9289
CD16+CD3+stroma	1.3062	0.4309
IDO+stroma	0.9692	0.6267
LAG3+stroma	0.9952	0.6158
TIM3+stroma	1.0031	0.7602
CD163+stroma	1.0011	0.8997
CD3+stroma	0.9949	0.7516
IDO+CD3-stroma	0.9437	0.5328
IDO+CD3+stroma	0.9711	0.8796

LAG3+CD3-stroma	0.9952	0.6688
LAG3+CD3+stroma	0.9574	0.4316
TIM3+CD3-stroma	1.0052	0.7324
TIM3+CD3+stroma	1.0045	0.8525
CD163+CD3-stroma	1.0005	0.9608
CD163+CD3+stroma	1.0075	0.7821
IDO+CD163-stroma	0.9806	0.8610
IDO+CD163+stroma	0.9025	0.4435
LAG3+CD163-stroma	0.9950	0.6874
LAG3+CD163+stroma	0.9800	0.5280
TIM3+CD163-stroma	0.9795	0.6663
TIM3+CD163+stroma	1.0053	0.6445
IDO+LAG3-stroma	0.9744	0.7128
IDO+LAG3+stroma	0.4964	0.2188
IDO+TIM3-stroma	0.9774	0.8574
IDO+TIM3+stroma	0.9216	0.4860
LAG3+TIM3-stroma	0.9952	0.6744
LAG3+TIM3+stroma	0.9711	0.5009
LAG3-TIM3+stroma	1.0052	0.6203
LAG3-TIM3-stroma	1.0004	0.9575
IDO-LAG3+stroma	0.9954	0.6305
IDO-TIM3+stroma	1.0043	0.6915
IDO+LAG3-CD3+stroma	0.9856	0.9420
IDO+LAG3+CD3+stroma	0.0182	0.2161
IDO+TIM3-CD3+stroma	0.9720	0.9568
IDO+TIM3+CD3+stroma	0.9645	0.8755
LAG3+TIM3-CD3+stroma	0.9503	0.4749
LAG3+TIM3+CD3+stroma	0.8797	0.4685
LAG3-TIM3+CD3+stroma	1.0075	0.7612
IDO-LAG3+CD3+stroma	0.9579	0.4410
IDO-TIM3+CD3+stroma	1.0059	0.8230
IDO+LAG3-CD163+stroma	0.8987	0.4765
IDO+LAG3+CD163+stroma	0.2696	0.2639
IDO+TIM3-CD163+stroma	0.2335	0.1163
IDO+TIM3+CD163+stroma	0.9141	0.5455
LAG3+TIM3-CD163+stroma	0.9580	0.5069
LAG3+TIM3+CD163+stroma	0.9696	0.5866
LAG3-TIM3+CD163+stroma	1.0072	0.5465
IDO-LAG3+CD163+stroma	0.9810	0.5491
IDO-TIM3+CD163+stroma	1.0067	0.5859
LAG3+TIM3+IDO+stroma	0.2489	0.1895

LAG3+TIM3+IDO-stroma	0.9729	0.5331
LAG3+TIM3-IDO+stroma	0.2618	0.2652
LAG3-TIM3+IDO+stroma	0.9259	0.5483
LAG3+TIM3-IDO-stroma	0.9953	0.6824
LAG3-TIM3+IDO-stroma	1.0065	0.5580
LAG3-TIM3-IDO+stroma	0.9933	0.9605
LAG3+TIM3+IDO+CD3+stroma	0.0129	0.2190
LAG3+TIM3+IDO-CD3+stroma	0.8861	0.5007
LAG3+TIM3-IDO+CD3+stroma	0.0000	0.4554
LAG3-TIM3+IDO+CD3+stroma	0.9828	0.9430
LAG3+TIM3-IDO-CD3+stroma	0.9502	0.4752
LAG3-TIM3+IDO-CD3+stroma	1.0093	0.7313
LAG3-TIM3-IDO+CD3+stroma	0.9815	0.9716
LAG3+TIM3+IDO+CD163+stroma	0.1886	0.2514
LAG3+TIM3+IDO-CD163+stroma	0.9723	0.6199
LAG3+TIM3-IDO+CD163+stroma	0.0034	0.3320
LAG3-TIM3+IDO+CD163+stroma	0.9158	0.5940
LAG3+TIM3-IDO-CD163+stroma	0.9587	0.5139
LAG3-TIM3+IDO-CD163+stroma	1.0086	0.4952
LAG3-TIM3-IDO+CD163+stroma	0.1973	0.1124

A HR >1 indicates an increased risk of death and HR <1 indicates a decreased risk of death.
**p*<0.05, ***p*<0.01, ****p*<0.001

Abbreviations: HR, hazard ratio

Table S4. Univariate Cox regression results for T cell exhaustion markers and checkpoint inhibitors.

**Marker**	**HR**	***p*-value**
LAG3+	1.00	0.62
TIM3+	1.00	0.76
IDO+	0.97	0.63
PD-1+	1.00	0.97
PD-L1+	1.00	0.77

A HR >1 indicates an increased risk of death and HR <1 indicates a decreased risk of death. The relative number of each immune cell type was measured in tumor-associated stroma.

Abbreviations: HR, hazard ratio
